# Straw Returning Methods Affects Macro-Aggregate Content and Organic Matter Content in Black Soils: Meta-Analysis and Comprehensive Validation

**DOI:** 10.3390/plants13233284

**Published:** 2024-11-22

**Authors:** Kangmeng Liu, Yu Hu, Yumei Li, Lei Wang, Liang Jin, Lianfeng Cai, Xiaoxiao Wu, Zhenguo Yang, Yan Li, Dan Wei

**Affiliations:** 1 College of Resources and Environment, Northeast Agricultural University, Harbin 150030, China; lkm0814@163.com (K.L.); huyu0805@126.com (Y.H.); clfcx13027878049@163.com (L.C.); wuxx_gsau@163.com (X.W.); yang11052950@163.com (Z.Y.); 2 Heilongjiang Academy of Black Soil Protection and Utilization, Harbin 150086, China; liyumeiwxyl@126.com; 3 Institute of Plant Nutrition, Resources and Environment, Beijing Academy of Agricultural and Forestry Sciences, Beijing 100097, China; wanglei@baafs.net.cn (L.W.); jinliang19762003@aliyun.com (L.J.)

**Keywords:** straw return, Northeast Black Soil Region, meta-analysis, organic matter, water-stable agglomerates

## Abstract

Straw returning into the soil is a crucial method for boosting soil carbon levels. To research the influence of straw return practices on soil aggregates and organic matter content within the farmlands of the Northeast Black Soil Region, the objective was to clarify the varying impacts of these practices on soil carbon enhancement. In this study, 89 pertinent papers were acquired through a rigorous literature compilation. Meta-analysis and the linear regression method were employed to analyze the influence of field return methods, their duration on soil water-stable aggregates, and their organic matter content. Furthermore, the study delved into the trends in the variation of aggregates and organic matter in relation to mean annual temperature and precipitation. Our results showed that the straw-returning method has been discovered to predominantly bolster soil organic matter by altering the proportions of macro-aggregate content. Specifically, straw incorporation has led to a notable enhancement in the content of macro-aggregates (57.14%) and micro-aggregates (20.29%), in addition to augmenting the content of macro-, small, and micro-aggregate organic matter by 13.22%, 16.43%, and 15.08%, respectively. The most significant increase in large agglomerates was witnessed in straw return over a period of more than 5 years (115.17%), as well as shallow mixing return (87.32%). Meanwhile, the highest increase in the organic matter content of large agglomerates was recorded in straw return over 5 years (12.60%) and deep mixing return (8.72%). In the field validation experiment, a period of seven years of straw return significantly boosted the macro-aggregate content across various soil layers, ranging from 11.78% to 116.21%. Furthermore, among the various climatic factors, the primary determinants of disparities in study outcomes were the average annual temperature and average annual precipitation. Specifically, lower precipitation and higher temperatures were conducive to the enhancement of macro-aggregate formation and organic matter content.

## 1. Introduction

Returning straw to fields is an effective yield-enhancing measure to build up land fertility. Increasing exogenous organic carbon inputs, particularly by enhancing the proportion of straw returned to the fields, has emerged as a pivotal factor contributing to the swift augmentation of organic carbon in China’s farmland over the past four decades. The straw returning to the field mentioned here refers to returning the above-ground crop straw to the soil in situ after the crop is harvested [1]. Conservation tillage techniques, primarily comprising straw mulching and minimal tillage (or no-tillage), have been comprehensively implemented across the black soil region in Northeast China [2,3], increasing organic matter content by 10 percent and effectively reducing surface runoff by a significant range of 40 to 80 percent [4,5]. In 2023, the extent of straw mulching and conservation tillage practices in the black soil region of Northeast China surpassed 195 million mu [6,7,8,9]. The research pertaining to straw incorporation into agricultural fields primarily centers on soil texture analysis, aggregate formation, carbon sequestration capabilities, enzyme activity assessment, and microbial population dynamics [10,11,12,13]. Straw farming effectively enhances soil properties in several pivotal ways. It lessens soil bulk weight, elevates soil porosity, and bolsters water and fertilizer retention capacity. Furthermore, it augments soil microbial diversity and enzyme activity, fostering the development of larger soil aggregates. Ultimately, straw farming significantly increases soil organic matter, leading to a healthier and more fertile soil ecosystem [14,15]. Inadequate farming practices can result in detrimental consequences, including the loosening of soil surfaces, soil weathering, and the exacerbation of crop diseases, among others [16].

Soil organic matter and aggregates serve as crucial indicators of soil fertility [17]. A favorable aggregate composition effectively maintains the loose and porous structure of the soil [18,19], while the content of organic matter influences both the formation and stability of these aggregates. Furthermore, these two factors mutually influence and restrict each other [20]. It has been discovered that straw mulching can notably enhance the content of macro-agglomerates while significantly diminishing the presence of micro-agglomerates [21,22]. However, contrasting findings have emerged from some scholars [23,24]. Consequently, the influence of straw mulching on soil may be contingent upon various factors, including soil type, the quantity of mulching applied, climate, and others. Secondly, there exist distinct variations in the impact of years of straw incorporation on soil fertility. Research scholars have categorized these years into three distinct periods: short-term (≤3 years), medium-term (3–5 years), and long-term (>5 years) and there exist notable differences in the impacts of varying years of return on soil properties [25,26,27]. Short-term straw returning leads to a swift accumulation of soil organic matter, yet this increase diminishes subsequent to a single growing cycle [28]. Long-term straw returning significantly enhances soil structure and function, leading to a substantial accumulation of organic carbon in the topsoil layer. This practice not only sustains land productivity, but also effectively mitigates the issue of nutrient depletion that often arises from short-term straw returning methods. As a result, the quality of black soil undergoes marked improvement [12]. At the same time, soil aggregates and organic matter content are also affected by climatic conditions, environmental background values, and other factors [29].

Meta-analysis represents a statistical approach to quantitatively integrating multiple independent research studies. In recent times, it has garnered significant attention and application within the realm of agronomy. Previous comprehensive evaluations have thoroughly examined the global-scale impacts of straw incorporation on various aspects, including crop growth, soil attributes, soil carbon sequestration, and nitrogen cycling [30,31,32,33,34,35]. Due to the unique soil background values and abundant crop straw resources in the black soil region, the northeastern area implements a black soil protection and utilization model, centered on straw incorporation as a primary measure. This approach is tailored to varying soil types, erosion levels, and other factors, aiming to achieve conservation, cultivation, carbon sequestration, fertilizer retention, erosion control, and obstacle mitigation, all in accordance with local conditions. Currently, there exists a scarcity of comprehensive assessments examining the impacts of straw incorporation on soil aggregates and their associated organic matter content within the black soil region of Northeast China. To delve deeper into the impacts of straw incorporation on carbon sequestration within soil aggregates in the black soil region, the current research employs meta-analysis to scrutinize alterations in the properties of water-stable aggregates and their organic matter content in relation to the duration and mode of straw incorporation. Additionally, it aims to elucidate the mechanism through which straw incorporation enhances the stability of aggregates and their organic matter content in the black soil region. The optimal strategies for incorporating straw back into the field were clarified, and the precision of the meta-analysis outcomes was corroborated through field trials, thereby furnishing a robust scientific foundation for the rational enhancement of soil texture and fertility.

## 2. Results and Analyses

### 2.1. The Impact of Straw Incorporation on Water-Stable Aggregates and Soil Organic Matter Content

**Figure 1:**
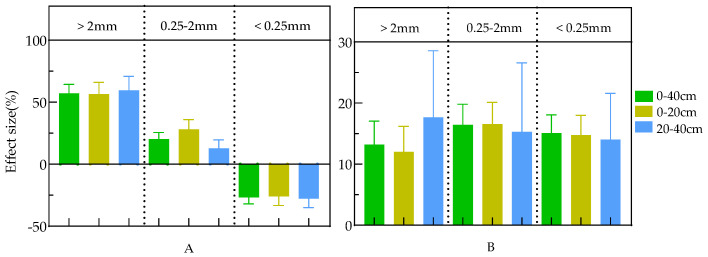
Effects of straw returning to field on water-stable aggregate (**A**) and organic matter (**B**) content.

The findings of this study are depicted in Figure 1. When straw is returned to the field as opposed to being left in the field, it significantly elevates the levels of water-stable large agglomerates (LA) and small agglomerates (SA), with respective increases of 57.14% and 20.29% (*p* < 0.05). Furthermore, there is a notable 26.74% decrease in the content of water-stable micro-agglomerates (MA). Additionally, the content of the organic matter in soil aggregates also significantly increases, with the following order of magnitude: SOM_SA_ (16.43%) > SOM_MA_ (15.08%) > SOM_LA_ (13.22%). The polysaccharides and organic acids, which are metabolites generated during the decomposition of straw when returned to the field, serve as direct cementing agents [24,36], stimulating soil microbial activities, accelerating the decomposition of organic matter, and enhancing the organic matter content within aggregates of various particle sizes [37,38]. As the soil layer deepens, the LA content exhibits a rising tendency, whereas the SA content reveals a declining trend. The MA content undergoes minimal change, and the SOM content follows a similar trend pattern.

### 2.2. The Influence of Return Method and Return Age on Water-Stable Agglomerate Content

**Figure 2:**
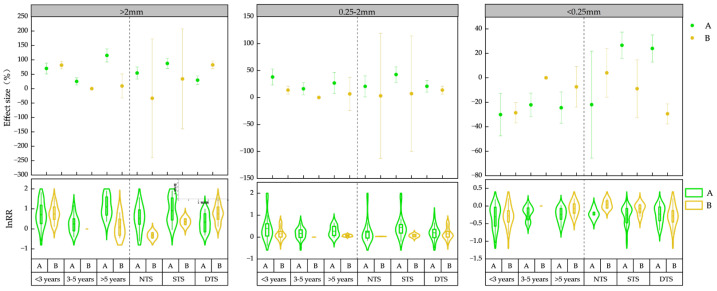
Effect of straw returning method (NTS, STS, and DTS) and age (<3 years, 3–5 years and >5 years) on water-stable aggregate contents in different soil layers (A and B). NTS, mulching return; STS: shallow-mixed return; DTS, deep-mixed return; <3 years, short-term; 3–5 years, medium-term; >5 years, long-term; A, 0–20 cm; B, 20–40 cm.

**Figure 3:**
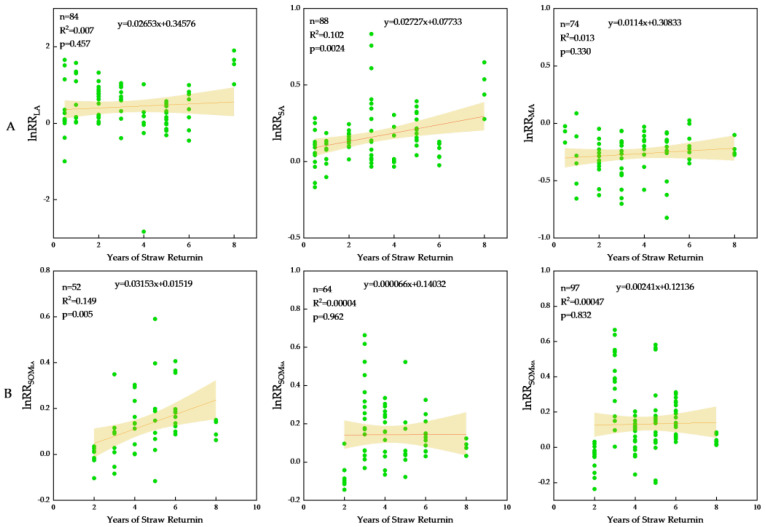
Relationship between water-stable aggregate content (**A**), organic matter content of aggregates (**B**), and straw returning years. The green dots represent response ratio of water-stable aggregate contents observations. The 95% confidence interval is indicated by a light brown shaded.

The analysis of the impact of straw returning years on the agglomerate content revealed (Figure 2) that, within the 0–20 cm soil layer, the magnitude of LA enhancement follows a pattern: >5 years yielding the highest increase (115.17%), <3 years showing a moderate increase (69.96%), and 3–5 years exhibiting the least significant rise (25.06%). Conversely, for SA, the order of increase was <3 years (38.01%), >5 years (26.82%), and 3–5 years (16.20%). As for MA, its content was reduced, with <3 years experiencing the largest decrease (30.08%), followed by >5 years (24.46%), and 3–5 years registering the lowest decrease (22.1%). Significant enhancement of LA content was observed under long-term straw return. As soil depth increased, short-term straw return revealed a notable rising trend in LA, while SA and MA exhibited a declining trend. This aligns with findings that the subsurface layer (20–40 cm) contains lower levels of SA and MA compared to the surface layer (0–20 cm), as previously documented [39]. Furthermore, straw field return enhanced LA content in the subsurface layer while reducing SA and MA content. A comprehensive analysis indicated that over time, with increased years of field return, LA and SA follow an initial increase before declining, whereas MA exhibits an opposite trend. Linear regression results (Figure 3A) reveal a positive correlation between agglomerate content and years of straw return.

In the 0–20 cm soil layer (Figure 2), the return method significantly impacted the LA content, with shallow-mixed return (STS) exhibiting the highest effect at 87.32%, followed by mulching return (NTS) at 54.27% and deep-mixed return (DTS) at 29.33%. Similarly, for SA content, STS surpassed DTS and NTS with 42.58%, while DTS contributed 20.78% and NTS just 20.61%. Notably, NTS had no substantial influence on MA content. However, in terms of MA content, both STS (23.99%) and DTS (26.56%) elicited an increase. Moving to the 20–40 cm soil layer, DTS displayed a contrasting pattern, decreasing MA content by 29.42% while simultaneously enhancing LA and SA content by 82.47% and 14.78%, respectively. In summary, all three return methods, STS, DTS, and NTS, positively influenced LA content. Additionally, both STS and DTS were effective in augmenting both SA and MA contents, underscoring their potential benefits in soil nutrient management.

### 2.3. The Impact of Return Method and the Age of Return on the Organic Matter Content of Water-Stable Aggregates

**Figure 4:**
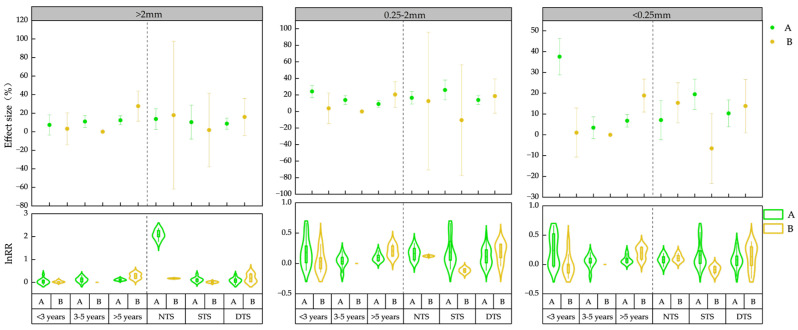
Effects of straw returning methods (NTS, STS and DTS) and years (<3 years, 3–5 years, and >5 years) on the content of organic matter in water-stable aggregates in different soil layers (A and B). NTS, mulching return; STS: shallow-mixed return; DTS, deep-mixed return; <3 years, short-term; 3–5 years, medium-term; >5 years, long-term; A, 0–20 cm; B, 20–40 cm.

Straw incorporation into the field significantly boosted the organic matter content within soil aggregates and enhanced the carbon sequestration capacity of larger aggregates [40]. Moreover, there existed variations in the distribution of organic matter among aggregates of varying grain sizes [41]. Within the 0–20 cm soil layer (Figure 4), field returns exceeding 3 years markedly elevated SOM_LA_ content (95%CI > 0), with the highest increase observed after >5 years (12.36%), followed by 3–5 years (11.00%). The increase in SOM_SA_ content followed a pattern of <3 years (24.2%), 3–5 years (13.86%), and >5 years (9.02%), while the SOM_MA_ content increased most prominently within <3 years (37.59%), followed by 5 years (6.76%). In the 20–40 cm soil layer, field returns of over 5 years significantly increased SOM_LA_ (27.60%), SOM_SA_ (20.54%), and SOM_MA_ (18.88%). Notably, the SOM_LA_ content of straw returned for >5 years exhibited an upward trend with soil depth (Figure 4). Linear regression analysis confirmed a significant positive correlation between the response of SOM_LA_ content to straw returning and the duration of straw return (Figure 3B) (*p* < 0.05). In conclusion, the practice of returning straw to the field over an extended period resulted in the most significant augmentation of SOM_LA_ content. As the duration of straw return increased, a notable upward trend in SOM_LA_ content was observed, whereas the contents of SOM_SA_ and SOM_MA_ exhibited a discernible downward trend.

In the 0–20 cm soil layer (Figure 4), both NTS (13.71%) and DTS (8.72%) demonstrated a marked elevation in content (95%CI > 0). Additionally, STS (25.98%), NTS (16.48%), and DTS (13.84%) significantly augmented the SOM_SA_ content (95%CI > 0), while STS (19.48%) and DTS (10.32%) contributed to an increase in SOM_MA_ content. Shifting to the 20–40 cm soil layer, both NTS and DTS significantly increased the SOM_MA_ content. Notably, it is apparent that DTS enhanced SOM_LA_ content as the sampling depth progressed.

### 2.4. The Impact of Additional Influencing Variables on Water-Stable Aggregates and Organic Matter Following Straw Incorporation into Agricultural Fields

**Figure 5:**
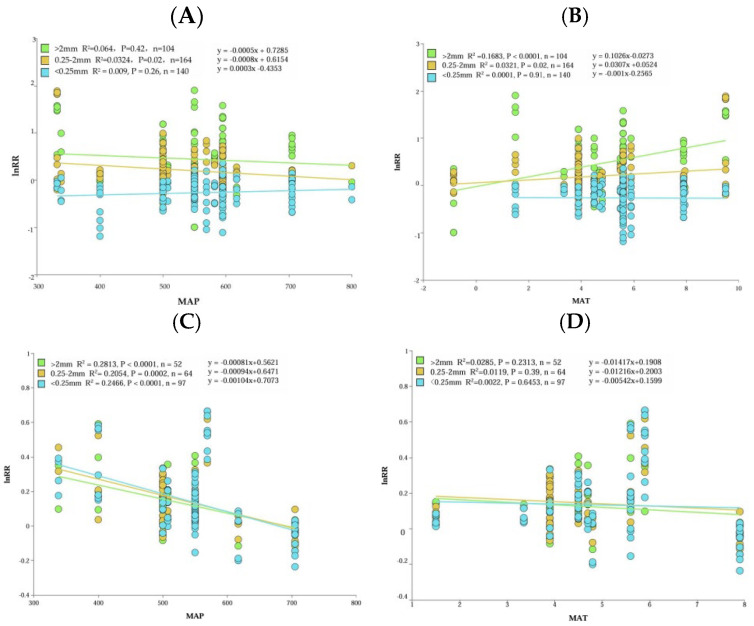
The relationship between water-stable aggregates (**A**,**B**), organic matter content (**C**,**D**), average annual precipitation (MAP), and average annual temperature (MAT).

Climatic factors significantly impact the input and decomposition of soil organic matter in agricultural soils [42]. Linear regression analysis revealed (Figure 5) that the influence of straw incorporation on LA and SA content exhibited a negative correlation with the rise in mean annual precipitation, while positively correlating with the content of MA. Notably, SA content underwent a marked decrease (*p* < 0.05) as mean annual precipitation increased, whereas LA content significantly increased (*p* < 0.05) with the elevation of annual average temperature. The content of agglomerates exhibited a positive correlation with the mean annual temperature, whereas the content of agglomerates’ organic matter displayed a negative correlation with both the mean annual temperature and mean annual precipitation. Notably, there was a significant decreasing trend observed in the content of agglomerates’ organic matter as the mean annual precipitation increased (*p* < 0.05), with less influence from the mean annual temperature. Returning straw to the field helped to reduce water evaporation and increase soil water content and temperature [43,44]. Additionally, conditions of lower rainfall and higher temperature promoted microbial and enzyme activities within the soil environment, accelerating straw decomposition and thereby boosting the content of agglomerates and their organic matter, as per reference [25]. In summary, these climatic conditions of reduced rainfall and elevated temperatures were conducive to the increased presence of LA and SOM_LA_ content.

### 2.5. The Correlation Between Water-Stable Agglomerate Response Ratios and Their Respective Organic Matter Response Ratios

**Figure 6:**
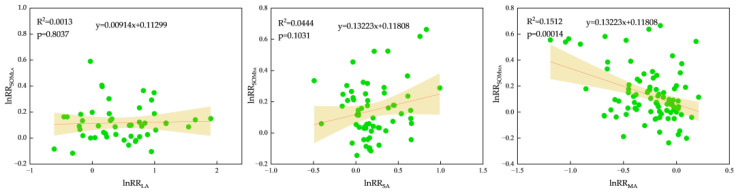
The relationship between the response ratio of aggregate and organic matter content.

The agglomerate development model postulates that substantial agglomerates emerge from the coalescence of smaller, independent agglomerates, united through the application of a binder. This structured formation offers superior safeguarding for the encapsulated organic carbon within [45]. To more clearly define the interplay between agglomerates and organic matter, we conducted an analysis focusing on the response ratios between agglomerates and organic matter content. Our findings (Figure 6) revealed that LA and SA exhibited positive correlations with the content response ratios of SOM_LA_ and SOM_SA_, respectively. Conversely, MA displayed a notable negative correlation with the content response ratio of SOM_MA_ (*p* < 0.05). Furthermore, we observed that as the particle size of agglomerates decreased, their organic matter content was also diminished.

### 2.6. Effects of Various Years and Return Methods on Soil Water-Stable Aggregates Content in Field Experiments

**Figure 7:**
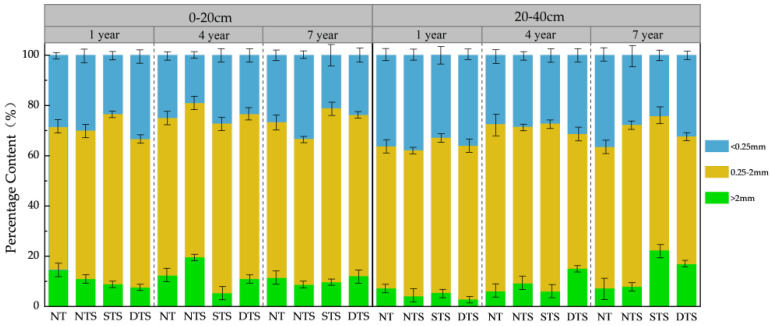
Effects of returning years (1 year, 4 years, and 7 years) and methods (NT, NTS, STS, and DTS) on soil water-stable aggregate (LA, MA and SA) content. NT, no tillage with straw removed from the field; NTS, no tillage with 100% straw retention; STS, tilling to a depth of 20 cm in autumn with 100% straw retention; DTS, tilling to a depth of 35 cm in autumn with 100% straw retention; LA, >2 mm; MA, 0.25–2 mm; SA, <0.25 mm.

In this research, a meta-analysis was conducted to evaluate the influence of the duration and method of straw incorporation on soil aggregates and their organic matter content. Subsequently, the findings were validated through the field experiment (Figure 7). When compared to straw removal from the field, incorporating straw for one year was observed to reduce the LA content by 36.39% in the 0–20 cm soil layer, while enhancing the SA content by 8.76% and MA content by 1.07%. This divergence from the meta-analysis outcomes can be attributed to several factors: Initially, soil microorganisms decompose the organic matter in straw, releasing nutrients. However, as the plant growth cycle concludes, microbial activity may diminish, leading to a slower rate of organic matter decomposition. Thus, the promotion of macroaggregate formation is reduced [46]. Four and seven years of straw return increased LA (2.91%, 11.78%) and SA content (3.36%, 3.00%) and decreased MA content (7.04%, 2.18%), and the results of medium- and long-term straw return were consistent with the results of the meta-analysis. Overall, the LA and SA contents consistently exhibited an upward trend as the years of straw return reincorporation increased, aligning with the linear regression outcomes. The MA content displayed a decreasing trend, which deviated from the study’s findings. This discrepancy can be attributed to the long-term straw reincorporation enhancing the soil structure and elevating the proportion of macro-aggregates. The formation of these larger aggregates may involve the incorporation or fusion of micro-aggregates, leading to a relative decrease in their content [46]. Secondly, the meta-analysis was conducted specifically in the Northeast Black Soil Region, yet it is important to note that the aggregate content of any particular test site is inherently influenced by various factors, including the soil’s background value, the application of fertilizers, and climatic conditions.

In the 20–40 cm soil layer, a one-year straw return significantly boosted LA content by 42.78% and SA content by 1.22%, while decreasing MA content by 10.54%. This observation aligns with the findings of the meta-analysis. The straw return of four years further increased LA content by 66.07% and MA content by 5.93%, but led to a reduction in SA content of 8.47%. After seven years of straw return, LA content soared by an impressive 116.21%, accompanied by a slight 0.10% increase in SA content and a substantial 23.22% decrease in MA content. However, these results diverged from the present study’s results, which can potentially be attributed to the effect of extended straw return years and deeper sampling depths, which fostered nutrient accumulation in lower soil layers, enhanced soil microbial activity, strengthened the aggregation capacity of soil particles, and ultimately increased the macro-aggregate content within the sub-tillage layer. Summarizing the findings, a seven-year straw return produced the most signific straw-derived organic particles, also known as colloids, which possess the capability to integrate with mineral matter, facilitating the coalescence of microaggregates into macroaggregates. This process enhances LA content while simultaneously diminishing MA content. The results show that 4-year and 7-year straw return, with the NTS and DTS methods, respectively, exhibited the most effective enhancement in LA content.

## 3. Discussion

### 3.1. The Impact of Straw Return Years on the Water-Stable Aggregate Content and Aggregate Organic Matter Composition

Straw-derived organic particles, also known as colloids, possess the capability to integrate with mineral matter, facilitating the coalescence of microaggregates into macroaggregates. This process enhances LA content while simultaneously diminishing MA content, as reported in studies [34,47]. Pei et al. [31] defined the global black soil region as the focus of their research. Due to geographical location and climatic factors, the impact of straw return on macroaggregate content was found to be insignificant. In this study, straw returning increased the contents of LA and SA; decreased the contents of MA; and significantly increased the contents of SOM_LA_ (16.43%), SOM_SA_ (15.08%), and SOM_MA_ (13.22%), which is consistent with the results of the meta-analysis of Sainan Geng et al. [34,39,47,48]. Li et al. [30] selected the literature after 2010 for meta-analysis, and the result was that straw returning to the field increased the organic matter of large aggregates by 12.80%, 12.75%, and 12.80%, respectively. However, the literature selected in this paper came from the recent 20 years, so the results were different. In this study, in the 0–20 cm soil layer, it was observed that long-term straw return significantly elevated LA and SOM_LA_ contents compared to short- and medium-term returns. Both LA and SOM_LA_ contents demonstrated a positive response to straw return, with the magnitude of increase correlating positively with the number of years of field return (Figure 3). This finding aligns with the results of Huang Lu et al. [49,50,51,52], as it underscores the intricate process involving numerous enzymes, a diverse array of microorganisms, and the synergistic action of soil bacteria and fungi, all of which are essential for the efficient degradation of straw and the subsequent promotion of soil nutrient cycling. Straw returning to field significantly increased the abundance of bacteria in soil [53], mainly in the form of a significant increase in eutrophic populations (such as Proteobacteria and Bacteroidetes), which have high metabolic activity and reproductive capacity and are conducive to the degradation of cellulose and lignin in soil, increasing the content of soil organic matter and maintaining the stability of soil structure [54]. When the duration of straw return to soil was brief, the readily decomposable organic matter underwent mineralization and decomposition, with minimal observable changes in organic matter content. However, as the years of straw return increased, the recalcitrant materials remaining in the soil transformed into humus, a difficult-to-decompose substance, which impacted the diversity of the bacterial community and enhanced soil nutrient and organic matter levels [55,56]. Stable organic matter contains lignin and other aromatic compounds that are difficult to decompose. These complex organic substances usually require specific microorganisms or enzymes to break them down. Higher soil temperature and adequate moisture are key factors in accelerating the decomposition of stable organic matter [39]. Concurrently, the heightened secretion from crop roots augmented the organic cementing agents surrounding the soil, fostering the development of larger aggregates [57]. In addition, the contents of SOM_SA_ and SOM_MA_ exhibited declines in correspondence with the growing years of straw return (Figure 3B), echoing the findings reported by Yinan Li et al. [30]. This trend can be attributed to the potential adaptive shifts within the soil microbial community, as the accumulation of organic matter in the soil surpasses a critical threshold. Afterward, the microbial populations specialized in straw decomposition may diminish. It is mainly some types of bacteria that specialize in breaking down cellulose and lignin that decline, which leads to a reduction in the rate of straw decomposition and conversion [58,59]. At the same time, the long-term practice of returning straw to the field significantly enhanced the soil structure and facilitated the formation of larger agglomerates. This improvement in soil structure led to an increase in the proportion of macroaggregates, specifically the SA and MA fractions, which became enveloped within even larger structures [51,60]. Consequently, there was a reduction in the content of SOM_SA_ and SOM_MA_.

In the 20–40 cm soil layer, short-term straw return had a pronounced impact on the content of aggregates, while long-term straw return significantly enhanced the organic matter content across all grain sizes of aggregates. As the soil layer deepened, the LA content of short-term straw return increased, whereas the SA and MA contents decreased. The long-term effect of straw return on the contents of LA, SA, and MA gradually diminished, while the augmentation of SOM_LA_ content displayed a positive upward trend. As plant roots descended, they penetrated and intertwined with soil particles, fostering the development of agglomerates [61]. Furthermore, microbial activities facilitated the continuous transfer of surface-layer-generated organic matter to deeper soil layers [62]. The combination of root secretions and organic matter enhanced the creation of larger aggregates in these deeper soils, thereby improving their carbon sequestration capacity [48]. Field validation has further substantiated the conclusion that a seven-year straw return program yields the most optimal impact on the LA content across both soil layers, aligning seamlessly with the findings of the meta-analysis.

### 3.2. The Impact of Straw Return Methods on the Water-Stable Aggregate Content and Aggregate Organic Matter Composition

Straw ploughing into the field markedly enhanced the abundance of large aggregates while exerting minimal influence on smaller aggregates. Furthermore, it significantly boosted the organic matter content within these large aggregates, albeit to a lesser extent compared to straw mulching [46,63]. It was also observed that straw plowing effectively reduced the LA content and enhanced the proportion of SA and MA, whereas the soil organic matter exhibited a decline in conjunction with the reduction in agglomerate size [64,65]. The enhancement of soil organic matter content achieved through straw ploughing into the field surpassed that accomplished by mulching [66]. In this study, within the 0–20 cm soil layer, STS exhibited the greatest enhancement of LA content, with NTS following closely behind. This finding contrasts with previous research by Wang Dandan et al.; specifically, NTS significantly augmented LA content [67], likely due to the fact that STS fosters the integration of straw into the fully tilled soil [68] while also minimizing soil disturbance, thereby bolstering aggregate stability and subsequently elevating LA content [69]. Both STS and DTS notably increased MA content, aligning with the findings reported by Hongjiu Yu et al. [64]. This is attributed to the soil structure damage caused by tillage depth and frequency, which promotes the fragmentation of large aggregates and their subsequent transformation into smaller aggregates. However, the carbon fixation capacity of micro-aggregates was weak, leading to the deterioration of soil structure, a reduction in aggregate stability, an increase in soil bulk density, a decrease in soil porosity and water content, an increase in organic matter exposure, and an acceleration of the mineralization rate [62]. The organic carbon content and stability of aggregates were reduced. The optimal enhancement of SOM_LA_ content was accomplished by NTS as a result of the formation of LA within NTS through organic cementation, leading to the creation of larger agglomerates. During this process, these agglomerates encapsulated a greater amount of carbon [70], ultimately resulting in an increase in the SOM_LA_ content.

In the soil layer spanning 20–40 cm, DTS significantly enhanced the level of LA content, whereas only NTS displayed a notable increase in SOM_MA_ content. As the sampling depth grew, the DTS facilitated a gradual rise in LA content, while NTS conspicuously augmented the SOM_MA_ content, aligning with the findings reported by Yiping Xu et al. [68]. DTS incorporated straw organic matter into the deeper soil layers, disrupted the plow pan, and augmented the soil’s aeration and water retention abilities, thereby fostering the development of LA in these deeper layers [71]. NTS significantly enhanced the SOM_MA_ content, as the straw organic matter primarily accumulated in the soil’s surface layer, while the deeper soil layers remained relatively unscathed by microbial activities, fostering the stabilization of SOM_MA_ [72].

### 3.3. Impact of Climatic Factors on Water-Stable Agglomerates and Organic Matter, and Their Interrelated Response Relationship

Temperature and precipitation represent pivotal climatic factors that significantly impact the stability of soil aggregates [59]. It was found that, due to the high multiple cropping index of farmland in North China, the organic matter supplemented by straw returning to the field would be consumed due to high intensity utilization, thus reducing the positive effect of soil aggregate carbon under the condition of straw returning to the field [26]. Abundant rainfall in Southwest China increases soil water content, creates suitable environmental conditions for microorganisms, and helps to stabilize ground temperature, which is conducive to the formation of humus and aggregates through decomposition of straw. It also increases the positive effect of carbon in soil aggregates. However, heavy rainfall increases the loss of soil nutrients [30]. Northeast China belongs to the temperate continental monsoon climate, and the soil is silty loam with good texture and strong water and fertilizer retention [51]. This special soil background value condition is more conducive to the increase in soil macroaggregate carbon. This research reveals a negative correlation between LA and its organic matter content with the mean annual precipitation. Conversely, LA displays a positive correlation with the average annual temperature, whereas its organic matter content exhibits an opposing trend. Lower precipitation and elevated temperatures were conducive to enhancing LA and SOM_LA_ contents, aligning with the findings of Hongwei Ouyang, Lixiao Ma et al. [43,44]. Given that moisture is a pivotal factor in the microbial decomposition of organic matter, under drought conditions, a decrease in soil moisture diminishes microbial activity, subsequently slowing down the decomposition of organic matter and facilitating the accumulation of organic matter within the aggregates. However, prolonged drought conditions can weaken plant roots and hinder microbial activities, potentially leading to the degradation of soil structure and an inability to sustainably maintain the stability of soil aggregates [73]. Therefore, it is of paramount importance to conduct a meticulous investigation into the impacts of varying mean annual precipitation and temperature levels on aggregates and their organic matter content. The study has revealed that as the particle size of soil aggregates diminishes, there is a corresponding decrease in the content of organic matter within them [41]. In this study (Figure 6), a clear positive correlation was observed between LA, SA, and the contents of SOM_LA_ and SOM_SA_, whereas a statistically significant negative correlation (*p* < 0.05) was noted between MA and SOM_MA_ content. The larger agglomerates, owing to their larger structures, effectively shield the internal organic matter, fostering a stable internal environment that mitigates microbial decomposition and enhances organic matter accumulation. Conversely, smaller agglomerates, characterized by a porous structure, are highly susceptible to external environmental perturbations. This leads to heightened microbial activity, thereby accelerating organic matter decomposition and limiting its adsorption capacity [74,75]. This study only analyzed the effects of straw returning to the field on soil aggregates and organic matter content, which provided a basis for future research on the effects of other organic materials returning to the field on soil fertility. Therefore, it is suggested to promote the long-term shallow mixture of straw to return to the field in the Northeast Black Soil area. In view of the slow decomposition rate of straw caused by low humidity and temperature in the Northeast Black Soil area, the research and development of microbial agents and soil water-retaining agent products were carried out, and the straw returning measures were combined not only to realize straw resource utilization and soil conservation, but also to improve soil fertility and carbon sequestration capacity.

## 4. Materials and Methods

### 4.1. Data Compilation

**Figure 8:**
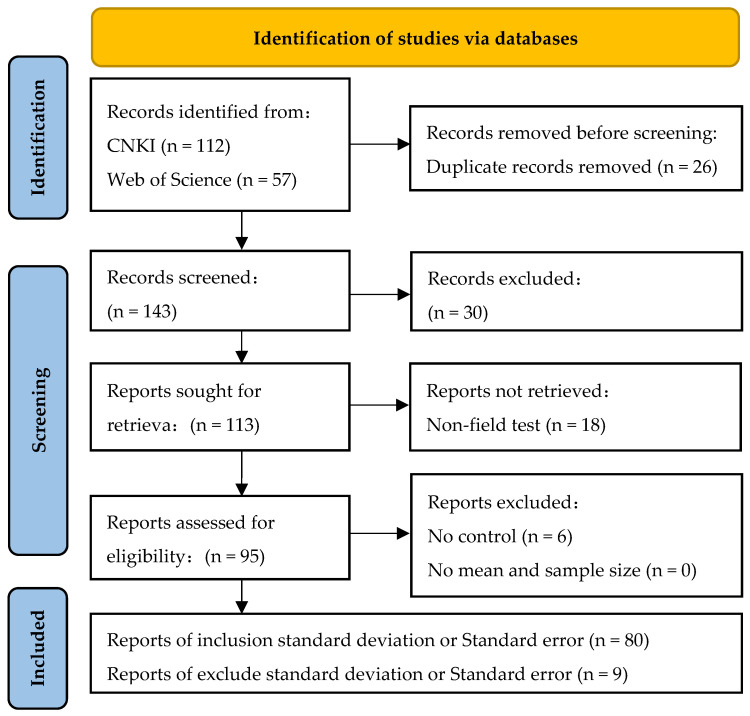
Flow diagram of database screening.

By utilizing both Chinese and English databases, including CNKI and Web of Science, we established the key theme words: (“black soil” or “Northeast” or “Heilongjiang” or “Jilin” or “Liaoning” or “Tongliao” or “Hulunbeier” or “Xinganmeng”) and (“Straw farming” or “Organic material farming”). There was no limit to the start time, and the end time was 1 January 2023. The exhaustive search for pertinent literature examined the impact of straw farming on soil parameters in the Northeast Black Soil Region. To mitigate publication bias, this study collected as much literature as possible related to the research topic. A comprehensive total of 89 articles (S1), encompassing 3347 data sets from eligible literature, were ultimately incorporated into the study, with the literature screening flow depicted in Figure 8.

The literature selection criteria encompassed the following: (1) The study area was restricted to the black soil region situated in Northeast China, specifically encompassing Heilongjiang, Jilin, Liaoning, as well as the four eastern leagues of Inner Mongolia (namely Chifeng, Tongliao, Hulunbeier, and Xinganmeng), (2) The trial encompassed a field test, with maize, soybean, or wheat serving as the cultivated crops. (3) The treatment group involved various methods of straw return to the field, including mulching, shallow mixing, and deep mixing, whereas the control group underwent straw removal. (4) The trial time, location, treatment, and trial results remained consistent, with data from various trial years at the identical trial site being preserved. Additionally, for the same literature, the location, treatment, and test results were kept constant while retaining the data from differing test years at the same test site. For the literature fulfilling the established criteria, we extracted pertinent background details, including the geographical location of the test site, soil background values, climatic conditions, and pertinent treatment information such as the methods employed for fertilizer application and straw return. Additionally, we gathered data on the mean and standard deviation of soil aggregates and their organic matter content under various treatment regimes, along with the number of test replicates conducted. The literature, which included organic carbon and standard errors, was preserved and subsequently transformed into organic matter and standard deviation [31,76].

### 4.2. Data Grouping

The return methods were categorized into three primary groups: mulching return (NTS), shallow-mixed return (STS), and deep-mixed return (DTS). Additionally, the return periods were segmented into short-term (<3 years), medium-term (3–5 years), and long-term (>5 years). The soil aggregate particle size was categorized into three distinct levels [30]: large aggregates (LA, >2 mm), small aggregates (SA, 0.25–2 mm), and microaggregates, along with powdered clay aggregates (MA, <0.25 mm). The organic matter contents present in large, small, and micro-agglomerates were denoted, respectively, by SOM_LA_, SOM_SA_, and SOM_MA_.

### 4.3. Statistical Analysis

In this study, the natural logarithmic formula for the response ratio is [77,78]:

(1) $$\ln{RR}_{i}=\ln X_{t}/X_{c}=\ln X_{t}-\ln X_{c}$$

In the formula, $X_{t}$ and $X_{c}$ represent the mean value of agglomerates or organic matter content of a given grain size for the treatment and control groups, respectively.

Variance and weighting formula [79]:

(2) $$v_{i}=\frac{{{SD}_{t}}^{2}}{\left(n_{t}\times {X_{t}}^{2}\right)}+\frac{{{SD}_{c}}^{2}}{\left(n_{c}\times {X_{c}}^{2}\right)}\;$$

(3) $$w_{i}=1/v_{i}\;$$

In the formula: ${SD}_{t}$ and ${SD}_{c}$ and $n_{t}$ and $n_{c}$ are the standard deviation and sample size of the treatment and control groups, respectively. $v_{i}$ is the between-case variance, and $w_{i}$ is the weight of the ith study.

The weighted response ratios and their standard errors and 95% confidence intervals are formulated as follows [80]:

(4) $$\ln RR=\sum \left(\ln{RR}_{i}\times w_{i}\right)/\sum w_{i}\;$$

(5) $$S_{\ln RR}=\sqrt{1/\sum w_{i}}\;$$

(6) $$95\%CI=\ln RR\pm 1.96\times S_{\ln RR}\;$$

In the formula, if 95%CI > 0, it is significantly increased. If 95%CI < 0, it is significantly reduced. If 95%CI contains 0, no significant effect is considered [80].

The conversion of effect values to a percentage variable (Z) was calculated as follows [81]:

(7) $$E=\left(e^{\ln RR}-1\right)\times 100\%\;$$

In this paper, data bias is quantified using the number of insecurity. If the number of insecurity is greater than a critical value (5n + 10, where n is the number of cases collected in the literature), it represents a high level of confidence in the data significance results, and there is no publication bias [82].

### 4.4. General Situation of the Experimental Field

Validation analyses were employed within a long-term positioning test field initiated in September 2016, situated in Wenchun Town, Mudanjiang City (44°60′ N, 129°58′ E). This locale boasts a continental monsoon climate, characterized by an average annual temperature of 6.1 °C and an average annual precipitation of 700~800 mm. The experiment comprised four distinct treatments: NT (no tillage with straw removed from the field), NTS (no tillage with 100% straw retention), STS (tilling to a depth of 20 cm in autumn with 100% straw retention), and DTS (tilling to a depth of 35 cm in autumn with 100% straw retention). These treatments were all in accordance with standard fertilizer application practices, and each was replicated three times, resulting in a total of 12 plots. The field management practices remained consistent, with the exception of the varying ploughing patterns employed. Before the experiment, the basic nutrient contents in the soil were as follows: total nitrogen 1.12 g·kg^−1^, alkali-hydrolytic nitrogen 101.55 mg·kg^−1^, available phosphorus 26.50 mg·kg^−1^, available potassium 130.28 mg·kg^−1^, organic carbon 10.95 g·kg^−1^, and pH 7.93.

Measurement Items and Methods: Sampling was conducted in 2017, 2020, and 2023, respectively, subsequent to the autumn harvest. To ensure purity, impurities were meticulously removed. Using a shovel, fresh soil samples were obtained from each plot, specifically targeting the 0–20 cm and 20–40 cm soil layers. These samples were preserved in aluminum boxes for further analysis. During the preservation and drying process, the utmost care was taken to prevent mechanical pressure-induced deformation of the soil. Subsequently, wet sieving [83] was employed to segregate the soil into three distinct particle size fractions: >2 mm, 0.25–2 mm, and <0.25 mm agglomerates. A portion of each sample underwent additional processing, wherein the agglomerates were washed in an aluminum box to remove the supernatant. Following this, the samples were dried, weighed, and analyzed to accurately calculate the percentage of agglomerate content for each particle size fraction [58]. The remainder of the sample was subjected to a 2 mm sieve and subsequently air-dried, and thereafter, the content of organic carbon in each aggregate was determined by oxidizing and utilizing the volumetric method of potassium dichromate externally heated at high temperature [84].

### 4.5. Data Processing

Excel 2021 served as the primary tool for data collection and organization, while Getdata Graph Digitizer 2.24 facilitated the acquisition of graphical data. Meta-analysis was executed utilizing Metawin 2.1, and for graphical representation, both SPSS 27.0 and Origin 2018 were employed.

## 5. Conclusions

A comprehensive meta-analysis examining the influence of straw returning on water-stable aggregates and organic matter content within the black soil region of Northeast China reveals that diverse straw incorporation techniques primarily enhance soil organic matter content by modulating the proportion of water-stable macro-aggregates. Overall, straw returning led to an augmentation in the water-stable macro-aggregate content within the soil. Specifically, shallow mixing straw return resulted in a notable increase of 87.32% in water-stable macro-aggregate content, whereas long-term straw returning demonstrated the most pronounced enhancement, reaching a significant increase of 115.17%. Furthermore, as the soil depth progressed, deep mixing straw return was observed to elevate the water-stable macro-aggregate content while concurrently diminishing the water-stable micro-aggregate content. Distinct approaches to straw returning markedly enhanced the organic matter concentration within soil aggregates. Furthermore, as soil depth increased, continuous deep tillage practices efficiently augmented the organic matter content within macro-aggregates. Lower precipitation and elevated temperatures favorably enhanced the accumulation of macro-aggregates and their organic matter content. Field trials have substantiated that prolonged (7-year) straw incorporation significantly boosts soil macro-aggregate content (11.78% to 116.21%), aligning with the conclusions drawn from the meta-analysis.

## Data Availability

Data are contained within the article and Supplementary Materials.

## Supplementary Materials

The following supporting information can be downloaded at: https://www.mdpi.com/article/10.3390/plants13233284/s1, Supplementary File S1: Article used in the meta-analysis, Refs. [85,86,87,88,89,90,91,92,93,94,95,96,97,98,99,100,101,102,103,104,105,106,107,108,109,110,111,112,113,114,115,116,117,118,119,120,121,122,123,124,125,126,127,128,129,130,131,132,133,134,135,136,137,138,139,140,141,142,143,144,145,146,147,148,149,150,151,152,153,154,155,156,157,158,159,160,161,162,163,164,165,166,167,168,169,170].
