# Supplementary material for: Straw Returning Methods Affects Macro-Aggregate Content and Organic Matter Content in Black Soils: Meta-Analysis and Comprehensive Validation
Source: Plants (Basel). 2024 Nov 22;13(23):3284. doi: 10.3390/plants13233284 (PMC11644226; doi:10.3390/plants13233284)
Supplement: Supplementary file 1 [file plants-13-03284-s001.zip › plants-3303624-supplementary.pdf]

Supplementary Materials

Articles included in the meta-analysis

[1] Sun, W Q.; Chen, B Y.; Wang, H J.; et al. Microbial Interactions and Roles in Soil Fertility in Seasonal Freeze-Thaw Periods under Different Straw Returning Strategies[J]. Agriculture,2021,11(8):779-779.

[2] Fan, W.; Wu, J.; Ahmed S, et al. Short-Term Effects of Different Straw Returning Methods on the Soil Physicochemical Properties and Quality Index in Dryland Farming in NE China[J]Sustainability,2020,12(7):2631.

[3] Fan, W.; Wu, J.; Li, J.; et al. Comparative effects of different maize straw returning modes on soil humus composition and humic acid structural characteristics in Northeast China[J]. Chemistry and Ecology,2018,34(4):355-370.

[4] Wang, Q.; Jia, S X.; Liang, A Z.; et al. Residue Return Effects Outweigh Tillage Effects on Soil Microbial Communities and Functional Genes in Black Soil of Northeast China[J]. Chinese Geographical Science,2023,33(4):679-692.

[5] Liu, Y L.; Gu, Y.; Wu, C S.; et al. Short-Term Straw Returning Improves Quality and Bacteria Community of Black Soil in Northeast China[J]. Original Research,2022,31(2):1869-1883.

[6] Mabagala S F. Geng, Y H.; Cao, C J.; et al. Effect of Silicon Fertilizer and Straw Return on the Maize Yield and Phosphorus Efficiency in Northeast China[J]. Communications in Soil Science and Plant Analysis,2021,52(2):116-127.

[7] Qiang, S.; Xu, Y.; Jun, M.; et al. Long-Term Effects of Straw and Straw-Derived Biochar on Humic Substances and Aggregate-Associated Humic Substances in Brown Earth Soil[J]. Frontiers in Environmental Science,2022,10

[8] Wang, S C.; Lu, C A.; Huai, S C.; et al. Straw burial depth and manure application affect the straw-C and N sequestration: Evidence from 13C &15N-tracing[J]. Soil & Tillage Research,2021,208104884-.

[9] Wang, L.; Wang, C.; Feng, F.; et al. Effect of straw application time on soil properties and microbial community in the Northeast China Plain[J]. Journal of Soils and Sediments,2021,21(9):1-13.

[10] Xu, Y.; Qiang, S.; Jun, Y.; et al. Successive corn stover and biochar applications mitigate N2O emissions by altering soil physicochemical properties and N-cycling-related enzyme activities: A five-year field study in Northeast China[J]. Agriculture, Ecosystems and Environment,2022,340

[11] Zhao, Z H.; Zhang, C Z.; Wang, H Y.; et al. The Effects of Natural Humus Material Amendment on Soil Organic Matter and Integrated Fertility in the Black Soil of Northeast China: Preliminary Results[J]. Agronomy,2023,13(3):794-794.

[12] Chen, N N.; Zhao, X.; Dou, S X.; et al. The Tradeoff between Maintaining Maize (Zea mays L.) Productivity and Improving Soil Quality under Conservation Tillage Practice in Semi-Arid Region of Northeast China[J]. Agriculture,2023,13(2):508-508.

[13] Li, J Q.; Ye, X H.; An, J.; et al. The more straw we deep bury, the more soil TOC will be accumulated: when soil bacteria abundance keeps growing[J]. Journal of Soils and Sediments, 2022, 22(1): 162-171. DOI:10.1007/s11368-021-03068-w.

[14] Gan, J.; Zou, W.; Han, X.; et al. Effects of Organic Materials and Their Incorporation

Depths on Humus Substances Structure and Soil Microbial Communities' Characteristics in a Chinese Mollisol[J]. Agronomy,2023,13(8):2169-.
[15] Ma, Q.; Jiang, C.; Li, S.; et al. Maize yield and nitrogen-use characteristics were promoted as consistently improved soil fertility: 6-year straw incorporation in Northeast China[J]. Plant, Soil and Environment,2021,67(7):
[16] Gan, G W.; Qiu, C.; Han, X Z.; et al. Effects of 10 Years of the Return of Corn Straw on Soil Aggregates and the Distribution of Organic Carbon in a Mollisol[J]. Agronomy,2022,12(10):2374-2374.
[17] Liu, X.; Dou, X.; Zheng, S. Effects of Corn Straw and Biochar Returning to Fields Every Other Year on the Structure of Soil Humic Acid[J]. Sustainability,2022,14(23):15946-15946.
[18] Yang, X.; Meng, J.; Lan, Y.; et al. Effects of maize stover and its biochar on soil CO2 emissions and labile organic carbon fractions in Northeast China[J]. Agriculture, Ecosystems and Environment,2017,24024-31.
[19] Cao, D Y.; Lan, Y.; Sui, Q.; et al. Maize straw and its biochar affect phosphorus distribution in soil aggregates and are beneficial for improving phosphorus availability along the soil profile[J]. European Journal of Soil Science,2021,72(5):2165-2179.
[20] Zhu, F N.; Lin, X X.; Guan, S.; et al. Deep incorporation of corn straw benefits soil organic carbon and microbial community composition in a black soil of Northeast China[J]. Soil Use and Management,2022,38(2):1266-1279.
[21] Wang, X Q.; Lyu, G Y.; Zhang, Y.; et al. Annual burying of straw after pelletizing: A novel and feasible way to improve soil fertility and productivity in Northeast China[J]. Soil or Tillage Research,2023,230
[22] Huang, D D.; Chen, X W.; Cao, G J.; et al. Effects of Long-Term Conservation Tillage on Soil Nitrogen Content and Organic Nitrogen Components in a Chinese Mollisol[J/OL]. Applied Ecology and Environmental Research, 2018, 16(5): 5517-5528. DOI:10.15666/aeer/1605_55175528.
[23] Zhang, Y L.; Sun, C X.; Wang, S Q.; et al. Stover and biochar can improve soil microbial necromass carbon, and enzymatic transformation at the genetic level[J]. GCB Bioenergy,2022,14(10):1082-1096.
[24] Ya, O.; Chen, X D.; Li, J M.; et al. Evaluation of the Continuous Application of Different Organic Materials on Soil Surface Charge and Chemical Properties Along the Soil Profile of a Typical Mollisol[J]. Original Research,2022;31(1):245-256.https://doi.org/10.15244/pjoes/138987
[25] Lian, H L.; Wang, Z Y.; Li, Y N.; et al. Straw Strip Return Increases Soil Organic Carbon Sequestration by Optimizing Organic and Humus Carbon in Aggregates of Mollisols in Northeast China[J]. Agronomy,2022,12(4):784-784.
[26] Qiao, Y.; Miao, S.; Zhong, X.; et al. The greatest potential benefit of biochar return on bacterial community structure among three maize-straw products after eight-year field experiment in Mollisols[J]. Applied Soil Ecology,2020,147103432-103432.
[27] Song, G.; Jia, S L.; Yue, R L.; et al. Soil organic carbon associated with aggregate-size and density fractions in a Mollisol amended with charred and uncharred maize straw[J]. Journal of Integrative Agriculture,2018,18(7):1496-1507.
[28] Shuang, Z.; Sen, D.; Hong, M D.; et al. Fluorescence Spectroscopy and C NMR Spectroscopy Characteristics of HA in Black Soil at Different Corn Straw Returning

Modes[J].International Journal of Analytical Chemistry,2021,20219940116-9940116.
[29] Qiu, C.; Han, X Z.; Chen, X.; et al. Effects of organic amendment depths on black soil pore structure using CT scanning technology[J]. Transactions of the Chinese Society of Agricultural Engineering (Transactions of the CSAE), 2021, 37(14): 98-107.
[30] Qi, Z J.; Song, F.; Zhang, Z G.; et al. Effects of different conservation tillage methods on soil hydrothermal condition as well as maize yield in cold black soil region[J]. Transactions of the Chinese Society for Agricultural Machinery, 2022, 53 (12): 380-389.
[31] Li, Y H.; Gu, S Y.; He, W Y.; et al. Effects of Conservation Tillage Practices on Organic Carbon Components and Maize Yield in Black Soil[J]. Chinese Journal of Soil Science, 2023, 54(2): 336 – 345
[32] Xu, Y Y.; Sun, S M.; Jin, X Y.; et al. Effects of Different Tillage Measures on Soil Texture and Maize Yield [J]. Journal of Maize Sciences,2022,30(04):97-106.DOI:10.13597/j.cnki.maize.science.20220413.
[33] Wang, C.; Wang, Y F.; Gu, X J.; et al. Effects of Continuous Application of Biochar on the Basic Physicochemical Properties of Black Soil [J]. Chinese Journal of Soil Science, 2018, 49(2): 428- 434
[34] Xu, Z B.; Li, L.; Wang, H B.; et al. Effects of different tillage methods on aggregate and organic carbon distribution in black soil[J/OL]. Journal of Jilin Agricultural University,1-9[2024-10-08].https://doi.org/10.13327/j.jjlau.2021.1767.
[35] Gao, H J.; Peng, C.; Zhang, X Z.; et al. Effects of different straw returning modes on characteristics of soil aggregates in chernozem soil[J]. Journal of Soil and Water Conservation,2019,33(01):75-79.DOI:10.13870/j.cnki.stbcxb.2019.01.013.
[36] Gao, H J.; Peng, C.; Zhu, M.; et al. Effects of Different Rotational Tillage Patterns on Soil Microbial Community Structure[J]. Journal of Maize Sciences,2021,29(05):104-112.DOI:10.13597/j.cnki.maize.science.20210514.
[37] Gao, H J.; Li, Q.; Peng, C.; et al. Effects of Different Crop Rotation and Straw Returning Methods on Bacterial Community Structure in Black Soil[J]. Journal of Jilin Agricultural University,2022,44(03):336-344.DOI:10.13327/j.jjlau.2021.1135.
[38] Gao, H J.; Peng, C.; Zhang, X Z.; et al. Effects of Corn Straw Returning Amounts on Carbon Sequestration Efficiency and Organic Carbon Change of Soil and Aggregate in the Black Soil Area[J]. Scientia Agricultura Sinica,2020,53(22):4613-4622.
[39] Liu, B D.; Chen, Y M.; Sui, Y Y.; et al. Effects of straw returning on nutrients and active organic carbon in black soil under different nitrogen application levels[J]. Soil and Fertilizer Sciences in China,2023,(02):10-15.
[40] Sun, J M.; Wu, J G.; Li, J M.; et al. Effects of different organic fertilizers on temporal-spatial distribution of nitrogen in black soil[J]. Journal of Soil and Water Conservation,2015,29(05):75-81.DOI:10.13870/j.cnki.stbcxb.2015.05.015.
[41] Chen, C.; Wu, J G.; Yang, Z Y. Effects of different manures and their mixed application on the dynamic changes of enzymes activity for black soil[J]. Journal of Soil and Water Conservation,2014,28(06):245-250.DOI:10.13870/j.cnki.stbcxb.2014.06.045.
[42] Li, J.; Chi, F Q.; Wei, D.; et al. Effects of different organic materials returning to file on the content of active organic carbon in black soil[J]. Soybean Science,2016,35(06):975-980.
[43] Cai, L J.; Zhang, J T.; Gai, Z J.; et al. Effect of the Amount of Stalk Return to Field on Soil Enzyme Activities under No- tillage[J]. Chinese Journal of Soil Science, 2015, 46(5): 1127-

1132
[44] Sa, R L.; Yang, H S.; Gao, J L.; et al. Effects of maize straw returning modes on soil fertility and maize yield[J]. Acta Agriculture Zhejiangensis,2018,30(02):268-274.
[45] Chen, Y.; Gao, J.; Zhu, P.; et al. Chemical characteristics of organic matter of different particle sizes and their response to long-term organic fertilization cultivation in the black soil of Northeast China[J]. Journal of Tianjin Normal University(Natural Science Edition),2022,42(01):59-65.DOI:10.19638/j.issn1671-1114.20220110.
[46] Li, N.; Long, J H.; Han, X Z.; et al. Effects of short-term plowing and organic amendments on soil physical properties and maize yield in dark brown soil in Northeast China[J]. Transactions of the Chinese Society of Agricultural Engineering,2021,37(12):99-107.
[47] Zou, W X.; Han, X Z.; Yan, J.; et al. Effects of incorporation depth of tillage and straw returning on soil physical properties of black soil in Northeast China[J]. Transactions of the Chinese Society of Agricultural Engineering,2020,36(15):9-18.
[48] Zou, W X.; Han, X Z.; Lu, X C.; et al. Effects of straw incorporated to different locations in soil profile on straw humification coefficient and maize yield[J]. Chinese Journal of Applied Ecology,2017,28(02):563-570.DOI:10.13287/j.1001-9332.201702.022.
[49] Cong, P.; Wang, J.; Dong, J X.; et al. Effects and analysis of straw returning on subsoil microbial community structure in black soil[J]. Transactions of the Chinese Society of Agricultural Engineering,2020,36(01):109-118.
[50] Jiao, L N.; Li, Z H.; Yin, C C.; et al. Effects of different stalk returned depth on soil humus and soil enzyme in black soil[J]. Soil and Fertilizer Sciences in China,2015,(02):17-21.
[51] Zhang, Z H.; Wu, S.; Zhai, C.; et al. Effects of Tillage and Straw Returning on Soil Physical Properties and Fixed Ammonium[J]. Journal of Maize Sciences,2019,27(03):102-107.DOI:10.13597/j.cnki.maize.science.20190316.
[52] Jiao, S.; Wang, W Y.; Zhao, X M.; et al. Effects of tillage methods on major fertility characteristics of chernozem soil and maize yield[J]. Agricultural Research in the Arid Areas,2020,38(01):31-38.
[53] Zhang, Y.; Xu, Z Q.; Xu, C L.; et al. Effects of fertility improvement measures on soil quality and maize yield in horizontal terraces fields of black soil hilly region[J]. Bulletin of Soil and Water Conservation,2022,42(06):190-196+222.DOI:10.13961/j.cnki.stbctb.2022.06.024.
[54] Wei, D.; Cai, S S.; Li, Y.; et al. The Response of Water-Soluble Organic Carbon to Organic Material Applications in Black Soil[J]. Scientia Agricultura Sinica,2020,53(06):1180-1188.
[55] Xu, Y P.; Tan, F.; Hu, Y P.; et al. Effect of Straw Returning on Cropland Soil Meso- and Micro-Arthropods Community in the Black Soil Area[J]. Chinese Journal of Zoology,2015,50(02):262-271.DOI:10.13859/j.cjz.201502012.
[56] Liu, P F.; Hong, M.; Chang, F.; et al. Impact of straw returning on cropland soil mesofauna community in the western part of black soil area[J]. Chinese Journal of Ecology,2018,37(01):139-146.DOI:10.13292/j.1000-4890.201801.001.
[57] Liu, P F.; Hong, M.; Mei, L.; et al. Impact of quantity of returned corn straw on the cropland ground arthropod community in a black soil area[J]. Acta Ecologica Sinica,2019,39(01):235-243.
[58] Liu, S J.; Guan, S.; Zhang, J J.; et al. Effects of Corn Straw Return on Aggregate-associated Organic Carbon Content in Black Soil: Based on Physical and Chemical Protection Supplied by Hierarchical Aggregates[J]. Journal of Jilin Agricultural

University,2019,41(01):61-70.DOI:10.13327/j.jjlau.2018.3730.
[59] Huo, H N.; Li, J.; Yuan, L.; et al. Effects of different straw returning amount on the potential gross nitrogen transformation rates of fertilized Mollisol[J]. Chinese Journal of Applied Ecology,2020,31(12):4109-4116.DOI:10.13287/j.1001-9332.202012.026.
[60] Lyu, Y J.; Yu, H Y.; Yao, F Y.; et al. Effects of soil straw return and nitrogen on spring maize yield, greenhouse gas emission and soil enzyme activity in black soils[J]. Chinese Journal of Eco-Agriculture,2016,24(11):1456-1463.DOI:10.13930/j.cnki.cjea.160405.
[61] Yan, L.; Li, S Y.; Meng, Q F.; et al. Effect of straw returning and organic manure on soil aggregate in black soil area [J]. Journal of Northeast Agricultural University, 2019, 50(12): 58- 67.DOI:10.19720/j.cnki.issn.1005-9369.2019.12.007.
[62] Yan, L.; Dong, T H.; La, Y P.; et al. Effects of no-tillage and straw returning on soil aggregates composition and organic carbon content in black soil areas of Northeast China[J]. Transactions of the Chinese Society of Agricultural Engineering,2020,36(22):181-188.
[63] Yan, L.; Zhou, L T.; Meng, Q F.; et al. Effect of organic materials returning on soil organic carbon concentration and soil organic carbon fractions in the black soil area[J]. Journal of Northeast Agricultural University, 2019, 51(5): 40-46.
[64] Fan, W.; Wu, J G.; Li, J M.; et al. Effects of Straw Return on Soil Physico-chemical Properties of Chernozem in Northeast China and Maize Yield Therein[J]. Acta Pedologica Sinica,2018,55(04):835-846.
[65] Cao, Q G.; Yang, F T.; Kong, F L.; et al. Total Straw Return with Strip-tillage Practices and Its Effect on Seeding Characters and Yield of Spring Maize[J]. Journal of Northeast Agricultural Sciences,2020,45(03):6-11.DOI:10.16423/j.cnki.1003-8701.2020.03.002.
[66] Dong, J X.; Cong, P.; Liu, N.; et al. Effects of Deep Straw Incorporation on Subsoil Physical Properties and Aggregate Distribution in Black Soil[J]. Acta Pedologica Sinica,2021,58(04):921-934.
[67] Yang, J J.; Gai, H.; Zhang, M X.; et al. Effect of Subsoiling Combined with Straw Returning Measure on Pore Structure of Black Soil[J]. Scientia Agricultura Sinica,2023,56(05):892-906.
[68] Cong, C.; Wang, T S.; Yue, L K.; et al. Amendment effect of subsoiling with organic materials application on soil physical properties of slope cropland in mollisol region[J]. Soil and Fertilizer Sciences in China,2021,(03):227-236.
[69] Dou, W S.; Dou, S. Effects of different returning methods of maize straw on surface and subsurface nutrients and humus in chernozem soil[J/OL]. Journal of Jilin Agricultural University,1-8[2024-10-08].https://doi.org/10.13327/j.jjlau.2021.1725.
[70] Liang, Y.; Cai, H G.; Yan, X G.; et al. Effect of Different Maize Straw-returning Modes on the Fertility of Black Soil[J]. Journal of Maize Sciences,2016,24(06):107-113.DOI:10.13597/j.cnki.maize.science.20160618.
[71] Liang, Y.; Cai, H G.; Yang, L.; et al. Effects of maize stovers returning by mulching or deep tillage on soil organic carbon sequestration in Mollisol[J]. Transactions of the Chinese Society of Agricultural Engineering[J]. Transactions of the Chinese Society of Agricultural Engineering,2021,37(01):133-140.
[72] Li, R P.; Luo, Y.; Sui, P X.; et al. Short-term effect of different returning methods of maize straw on the temperature of black soil plough layer[J]. Chinese Journal of Applied Ecology,2023,34(10):2693-2702.DOI:10.13287/j.1001-9332.202310.014.

[73] Zhang, H J.; Wang, S J.; Tian, C J.; et al. Effects of maize straw and its biochar on the dissolved organic matter characteristics of black soil in Northeast China[J]. Journal of Soil and Water Conservation,2021,35(02):243-250.DOI:10.13870/j.cnki.stbcxb.2021.02.032.
[74] Meng, W S.; Zhu, F N.; Zhang, B W.; et al. Effects of straw and biochar application on the soil physicochemical properties and corn yield in a black soil[J/OL]. Journal of Jilin Agricultural University,1-9[2024-10-08].https://doi.org/10.13327/j.jjlau.2023.20225.
[75] Zhang, S.; Yuan, Y H.; Yuan, B F.; et al. Effects of Maize Straw Returning with Deep Ploughing on Organic Carbon Content and Chemical Composition in Bulk Soil and Soil Aggregates[J/OL]. Journal of Jilin Agricultural University,1-14[2024-10-08].https://doi.org/10.13327/j.jjlau.2021.1111.
[76] Li, H.; Wu, J G.; Li, J M. Effects of strip composting for corn straw combined with livestock manure on soil active organic carbon[J]. Journal of Agro-Environment Science,2021 ,40(9):1944-1953.
[77] Li, C Y.; Yang, H H.; Sa, R L.; et al. Effects of straw returning on soil available nutrients and microbe biomass under different tillage methods[J]. Journal of Soil and Water Conservation,2017,31(01):197-201+210.DOI:10.13870/j.cnki.stbcxb.2017.01.033.
[78] Yan, H K.; Hu, B.; Gao, L Z. Effects on Available Nutrients of Brown Soil in Northern Liaoning under Long-term Use of Straw and Organic Fertilizer[J]. Journal of Shenyang Agricultural University,2013,44(06):812-815.
[79] Wang, M J.; Wang, F.; Su, S H, et al. Effects of Straw Turnover on Soil Water-Stable Aggregates and Soil Carbon Distribution[J]. Arid Zone Research,2019,36(02):331-338.DOI:10.13866/j.azr.2019.02.08.
[80] Zhang, J Q.; Li, F.; Sun, F C.; et al. Effects of different tillage methods on soil physical properties under the condition of straw returning[J]. Acta agriculture universitatis Jiangxiensis,2022,44(3):759-772.
[81] Wang, X Y.; Zhang, Y W.; Feng, Y.; et al. Effects of deep maize straw returning on soil fertility and maize yields[J]. Agricultural Research in the Arid Areas,2013,31(06):103-107.
[82] Ma, X Z.; Zhou, B K.; Zhang, X L.; et al. Effects of Conservation Cultivation Black Soil Physic-Chemical Properties in Arid Area[J]. Heilongjiang Agricultural Sciences,2009,(06):55-57+65.
[83] Zhao, W.; Chen, Y J.; Wang, H Y.; et al. Impact of Different Straw Return Systems on Nitrogen and Physical Characters in Black Soil[J]. Journal of Maize Sciences,2012,20(06):98-102.DOI:10.13597/j.cnki.maize.science.2012.06.001.
[84] Liu, J M.; Lu, P.; Xu, Y P.; et al. Effect of straw returning on soil organic carbon and soil nitrogen in black soil area of Jilin[J]. Soil and Fertilizer Sciences in China,2013,(03):96-99.
[85] Zhang, B.; He, H B.; Zhao, X X.; et al. Effects of Crop-residue Incorporation on No-tillage Soil Available Nutrients and Corn Yield[J]. Journal of Maize Sciences,2010,18(02):81-84.DOI:10.13597/j.cnki.maize.science.2010.02.023.
[86] Gao, H.; Wang, H Y.; Li, C B.; et al. Effects of Different Maize Straw Returning Modes on C/N Ratios in Mollisol[J]. Chinese Journal of Soil Science,2013,44(06):1392-1397.DOI:10.19336/j.cnki.trtb.2013.06.019.
[87] Zhang, Y.; Gao, Y.; Zhang, Y.; et al. Effects of residue return on stoichiometric characteristics of soil carbon, nitrogen, phosphorus, potassium, and the maize yield in black soil of Northeast China[J]. Journal of Plant Nutrition and Fertilizers,2023,29(01):31-

44.
[88] Lyu, F Z.; Yang, Y L.; Bao, X L.; et al. Effects of no-tillage and different stover mulching amounts on soil microbial community and microbial residue in the millisols of China[J]. Chinese Journal of Applied Ecology,2023,34(04):903-912.DOI:10.13287/j.1001-9332.202304.033.
[89] Gao, Y.; Liang, A Z.; Huang, D D.; et al. Effects of long-term no-tillage on the functional potential of microorganisms involved in the nitrogen, phosphorus and sulfur cycling of black soil[J]. Chinese Journal of Applied Ecology,2023,34(04):913-920.DOI:10.13287/j.1001-9332.202304.011.
